# Establishment of a patient-derived orthotopic osteosarcoma mouse model

**DOI:** 10.1186/s12967-015-0497-x

**Published:** 2015-04-30

**Authors:** Claudia Blattmann, Markus Thiemann, Albrecht Stenzinger, Eva K Roth, Anne Dittmar, Hendrik Witt, Burkhard Lehner, Eva Renker, Manfred Jugold, Viktoria Eichwald, Wilko Weichert, Peter E Huber, Andreas E Kulozik

**Affiliations:** Department of Pediatric Oncology, Hematology and Immunology, University of Heidelberg, Heidelberg, Germany; Department of Radiotherapy and Radiooncology, University of Heidelberg, Heidelberg, Germany; Division of Radiooncology, German Cancer Research Center (DKFZ), Heidelberg, Germany; Institute of Pathology, University of Heidelberg, Heidelberg, Germany; Division of Pediatric Neurooncology, German Cancer Research Center (DKFZ), Heidelberg, Germany; German Cancer Consortium (DKTK), Heidelberg, Germany; Department of Orthopedics, University of Heidelberg, Heidelberg, Germany; Core Facility, Small Animal Imaging Center, DKFZ, Heidelberg, Germany; National Center for Tumor Diseases (NCT), University of Heidelberg, Heidelberg, Germany

**Keywords:** Osteosarcoma, Xenograft, Patient-derived, Primary cell line, Mouse model

## Abstract

**Background:**

Osteosarcoma (OS) is the most common pediatric primary malignant bone tumor. As the prognosis for patients following standard treatment did not improve for almost three decades, functional preclinical models that closely reflect important clinical cancer characteristics are urgently needed to develop and evaluate new treatment strategies. The objective of this study was to establish an orthotopic xenotransplanted mouse model using patient-derived tumor tissue.

**Methods:**

Fresh tumor tissue from an adolescent female patient with osteosarcoma after relapse was surgically xenografted into the right tibia of 6 immunodeficient BALB/c Nu/Nu mice as well as cultured into medium. Tumor growth was serially assessed by palpation and with magnetic resonance imaging (MRI). In parallel, a primary cell line of the same tumor was established. Histology and high-resolution array-based comparative genomic hybridization (aCGH) were used to investigate both phenotypic and genotypic characteristics of different passages of human xenografts and the cell line compared to the tissue of origin.

**Results:**

A primary OS cell line and a primary patient-derived orthotopic xenotranplanted mouse model were established. MRI analyses and histopathology demonstrated an identical architecture in the primary tumor and in the xenografts. Array-CGH analyses of the cell line and all xenografts showed highly comparable patterns of genomic progression. So far, three further primary patient-derived orthotopic xenotranplanted mouse models could be established.

**Conclusion:**

We report the first orthotopic OS mouse model generated by transplantation of tumor fragments directly harvested from the patient. This model represents the morphologic and genomic identity of the primary tumor and provides a preclinical platform to evaluate new treatment strategies in OS.

## Background

Osteosarcoma (OS) is the most common malignant pediatric bone tumor. Standard therapy comprises neo-adjuvant chemotherapy, surgery and adjuvant chemotherapy with methotrexate, platin, alkylating agents and anthracyclines. While patients with localized and operable OS have a 5 year survival rate of approximately 60-70%, outcome for patients with metastatic disease or non-resectable tumors is poor [1]. Over the past 30 years, survival rates did not improve. The development of new therapeutic approaches is urgently needed. Therefore, valid preclinical models reflecting human osteosarcomas are crucially required to facilitate rapid and effective development of novel therapies.

In principle, animal models can either be generated by inducing a tumor in a model organism or by xenografting human cancer cells or tissue into immunodeficient mice [2]. For the development of novel treatment strategies, human tumor xenografts are currently the most widely used models in a preclinical setting. They most resemble the human tumor despite the restrictions due to the immunodeficiency of the host organism. Genetically engineered mouse models (GEM) allow the study of effects of inhibitors against defined molecular targets. With these increasingly sophisticated models tissue specific molecular changes can be compared between individual cancers and tissues on the molecular level. Non-germline genetically engineered models allow the analysis of the impact of specific cancer genes without some of the limitations inherent in traditional GEM models. As mechanisms of transformation and oncogenesis differ between “mice and men” the generation of clinically relevant models using the mouse requires their humanization [3].

GEM have been instrumental in understanding the molecular mechanisms involved in tumor initiation. However, they have been less successful in replicating advanced cancer. Moreover, a particular genetic alteration frequently leads to different tumor types in human and mouse and to lower metastatic rates in GEM than in humans. These shortcomings limit the capacity of current GEM models to predict clinical response to a particular therapy. In contrast, orthotopic xenografts of human tumors, or tumor cell lines, implanted in SCID mice have high rate of reproducibility [4].

In OS, animal models are difficult to establish: osseous tissue is difficult to handle mechanically and technically challenging to be xenografted orthotopically. Previously, subcutaneous xenografts and orthotopic OS mouse models have been described using injection of cell suspensions and commercially available human cell lines [5-7]. Furthermore, there have been studies reporting genetically engineered models as well as models that employ tumor self-seeding following injection of tumor cells into the blood circulation of nude mice [8-10]. However, the applicability of these models is limited because genetically manipulated tumor cells are used to establish such models.

Hence, our goal was to establish a novel preclinical platform for rapid and effective development of new treatment strategies. Our new model system allows the generation of an orthotopic mouse model and a corresponding OS cell line using patient derived primary OS tissue.

MR imaging and histopathology, as well as array-CGH analyses were used to compare progression and human tumor. In addition, array-CGH analysis proved the genetic stability of this model, which will be used for preclinical developing of new treatment strategies in the future.

## Material and methods

### Patients and tumor samples

Tumor tissue samples were obtained from a patient with relapsed high-grade OS [11]. The study was approved by the ethics committee of the Medical Faculty of the University of Heidelberg . After surgical resection and histopathological confirmation of the OS, the samples are stored in NaCl 0.9% on ice. A portion of the sample was xenotransplanted immediately (fraction 1, see below). The remaining part of the samples was split into three further fractions (fraction 2 to 4) as follows: fraction 2 was put into DMEM medium, 10% DMSO (Dimethylsulfoxide), 20% FCS (fetal calf serum) and 1% NEAA (Non Essential Amino Acid) under sterile conditions, was frozen stepwise to −80°C and then transferred to liquid nitrogen. Fraction 3 was snap frozen and stored in liquid nitrogen. The fourth fraction was brought into cell culture in DMEM medium, 10% FCS and 1% NEAA (Non Essential Amino Acid) under sterile conditions and was cultured under standard conditions.

### Establishment of xenografts

Fraction 1 of the human OS tissue was inserted into ten week old athymic BALB/c Nu/Nu mice (Charles River, Wilmington, Mass.) as follows:

Tumor samples were cut into 1 × 1 × 1 mm^3^ pieces. The right tibia of 6 mice was opened in the central (medial) part by drilling with a dental drill with a diameter of 0.5 mm to insert one tumor fragment in contact with the bone marrow. The anesthesia of mice was performed with isoflurane inhalation (1,5-3,5 Vol% per liter oxygen). After, the surgical wound was sutured. The whole procedure took about 10 min per mouse. The percentage of success was 92%. The mice were maintained under specific pathogen-free conditions, food and water were supplied ad libitum. Housing and all procedures involving the mice were performed according to the protocols approved by the German Cancer Research Center institutional animal care and use committee and by the local responsible government department (Regierungspräsidium Karlsruhe). Mice were observed daily for tumor growth by palpation and inspection.

**Figure 1:**
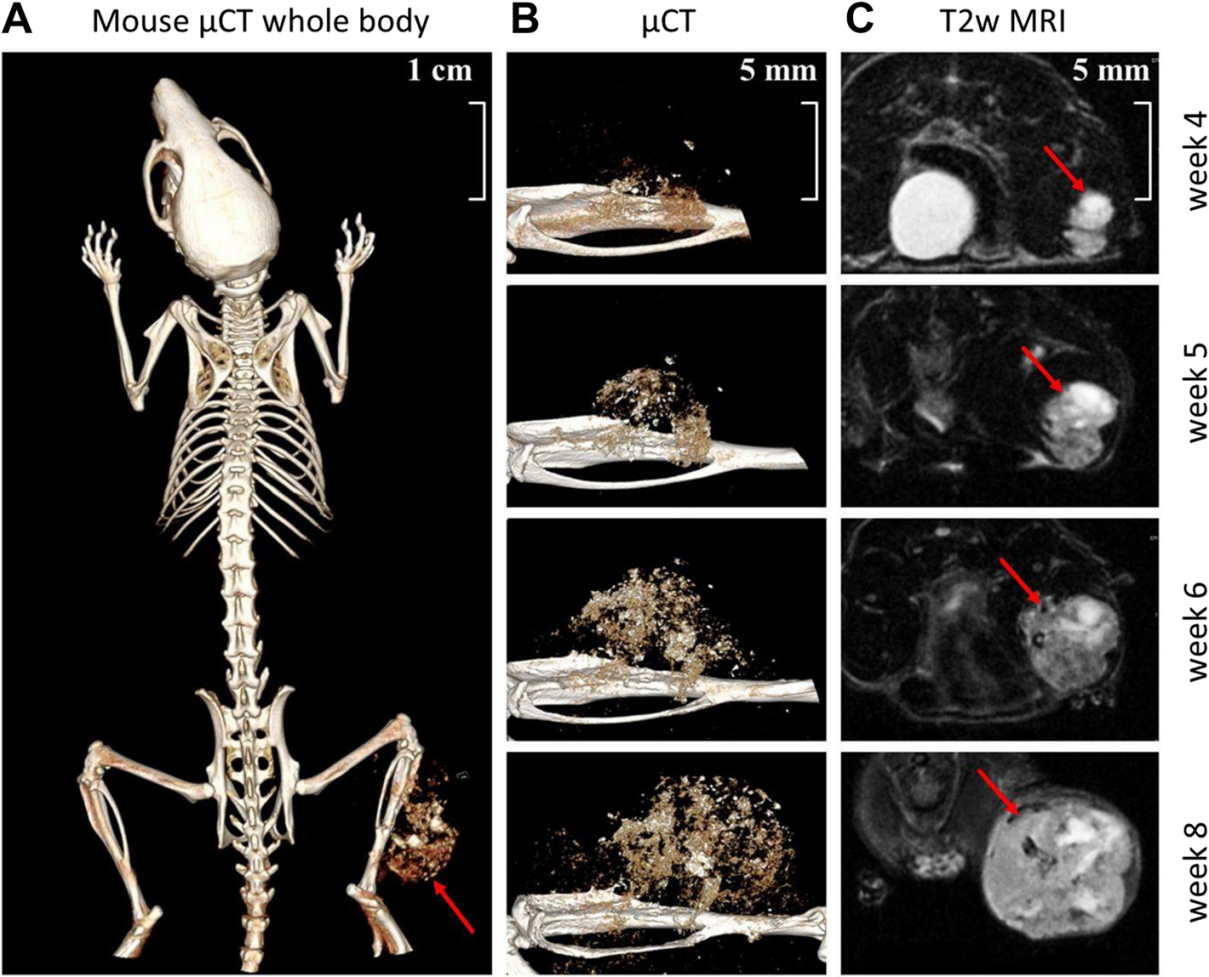
Representative MRI and μCT images of mouse xenografts 4 to 8 weeks after intratibial transplantation of patient-derived human OS tissue showing tibial tumor mass. **A)**: μCT image of whole mouse body 8 weeks after tumor inoculation. **B)** and **C)**: μCT and T2 weighed (T2w) MRI images of tumor growth from week 4 to week 8. Red arrows indicate tumor location.

MicroCT scans were acquired using an Inveon PET/SPECT/CT system (Siemens Medical Solutions, Knoxville, TN, USA). A 12 minute and 30 seconds CT scan was performed with parameter settings: 360 rotation steps, tube voltage 50 kV, tube current 500 μA, binning 1 and exposure time 400 ms. The pixel size was 0.0143 × 0.0143 × 0.0143 mm. Image reconstruction was performed using the conventional Inveon Research Workplace software 4.0. The reconstruction filter was Shepp-Logan with a downsample factor of 2. MRI was performed using a small animal MRI scanner (Bruker Icon, 1 Tesla, Ettlingen, Germany). Because sufficient contrast for tumor discrimination could not be achieved using i.p. injection of Magnevist (Bayer Schering Pharma) at a concentration of 0.5 mmol/ml for contrast enhanced T1w mri, tumor growth was visualized using T2w images (TE = 100 ms, TR = 2842 ms, FA = 180, voxel size = 0.16x0,16x1 mm^3^, FOV 30x30 mm, matrix size = 192x192, averages = 4). Lesion sizes were measured using T2 weighed (T2w) images and were found to match with tumor sizes obtained via μCT (Figure 1) and with weighed resected tumor tissues (data not shown). Measurements were performed by manual segmentation of the mass using Bruker ParaVision software. Mice were immobilized during the imaging procedures via Sevofluran inhalation narcosis at a dosage of 4% in air.

“Sufficient tumor growth” was defined as tumors in 3 of 6 mice attaining 1500 mm^3^. Each tumor was then removed and processed for the following analyses: (1) histopathological examination (2) array-based comparative genomic hybridization (aCGH) (see below), (3) orthotopic re-transplantation into 6 further mice, (4) re-cultivation in cell culture. One of the six tumors was cut into 1 × 1 × 1 mm^3^ fragments and transferred twice into 6 mice per passage (designated as passage P1 to P3). In the first passage (P1) three of six mice, in the second passage (P2) four of six mice and in the third passage (P3) six of six mice developed an orthotopic OS, which were frozen as outlined above for further analysis.

### Establishment of primary OS cell line

The original tumor tissue was collected and directly cultivated into medium (P1*). After 26 days, cells were split and 3.75 × 10^6^ cells were injected subcutaneously into the left flank of six athymic BALB/c Nu/Nu mice (Charles River, Wilmington, Mass.). After three weeks, four of six mice developed a tumor mass which was removed when reaching a volume of 1500 mm^3^. Tumor fragments were disaggregated and passaged again (P2*), split after 20 days and injected into the flank of six further mice. After two weeks, all mice developed a tumor mass, which was removed, disaggregated and re-passaged (P3*). Each resected xenograft was first disaggregated into a cell line and then cells were re-injected. The cell line “passages” means sequentially passaged cells subcutaneously through the mouse and not just in culture. The third passage was used for further analysis. Cells were tested and proved to be free of mycoplasma, viral as well as cell contamination using in-house Multiplex cell Contamination testing (McCT) service [12]. Genetic stability of the cells was compared to the original human tumor sample at each passage by aCGH (see below).

### Microarray-based comparative genomic hybridization (a CGH)

Genomic DNA from fresh frozen tissue was isolated using standard phenol-chloroform extraction. Genomic DNA from cultured cells was isolated using the Blood and Cell Culture Kit (Qiagen, Hilden, Germany) according to the manufacturer’s instructions. Selection of genomic clones, isolation of BAC DNA, performance of degenerate oligonucleotide primer-PCR, and preparation of microarrays were performed as previously described [13]. Labeling, hybridization, and washing procedure were performed as reported previously [14]. Array- (or matrix-) CGH was carried out as described, gains were defined as copy number imbalances log2 ratio > 0,25 and losses log2 ratio < −0,25 [15,16].

### Histopathology

Histology was performed nine weeks after tumor implantation into the mice. 3 μm thick whole tumor sections were cut from formalin-fixed, paraffin-embedded (FFPE) tissue blocks of all tumors. Sections were stained with hematoxylin-eosin (HE; Sigma-Aldrich, St Louis, USA). Histological comparisons were performed by two pathologists (AS, WW) using conventional HE-stainings as well as PAS and Masson Trichrom staining.

## Results

### Establishment of a primary osteosarcoma orthotopic mouse xenograft

Human OS tissue was collected from a 17 years old girl with an OS relapse in the right tibia with pulmonary and mediastinal metastases. Primary disease had been diagnosed two years previously with localized disease in the right distal femur. She received neo-adjuvant chemotherapy according to the EURAMOS 1 protocol [17]. The tumor was then completely resected and showed a grade 2 histological response (according to Salzer-Kuntschik; [18]). Subsequently, the patient received 3 courses of adjuvant chemotherapy. However, the protocol was discontinued by request of the patient and her family. The patient’s mother died of breast cancer at an age of 43 years and the younger brother died of embryonal rhabdomyosarcoma of the chest wall at an age of 12. The patient and her family did not consent to molecular diagnostic assessment to prove the presence of a familial tumor predisposition syndrome (Table 1).

**Table 1:** **Patient characteristics of tissue used for generation of patient-derived orthotopic mouse model**

**Age & Gender**	**17 years, female**
**Primary disease**	diagnosed April 2008
**- histopathology**	osteoblastic osteosarcoma
**- Tumor location**	right femur epiphysis, no metastases
**- Treatment**	according to EURAMOS 1 06/2008 – 11/2009, abortion of chemotherapy after the third postoperative cycle by request of the patient and her parents
	Extraarticular tumor resection of the right femur (09.09.2008) and implantation of a Mutars-Endoprothesis (regression grade II according to Salzer-Kuntschik)
**1. Relapse**	diagnosed 24 months after diagnose of primary disease
**- histopathology**	- osteoblastic osteosarcoma
**- Location**	- multifocal lesions in the right tibia, both lungs bilateral and the mediastinum
**- treatment**	- no further treatment because of the significant reduced general conditions and by request of the patient and her parents
**Death of disease**	8 weeks after diagnose of relapse
**Family history**	- Oldest child of four children
	- Youngest brother died because of an embryonal rhabdomyosarcoma in 2000
	- Mother died because of a Mamma-Ca in 2006
	- Father, one younger sister and one younger brother are healthy
	- Diagnostic concerning familiar tumor predisposition syndrome was declined

**Figure 2:**
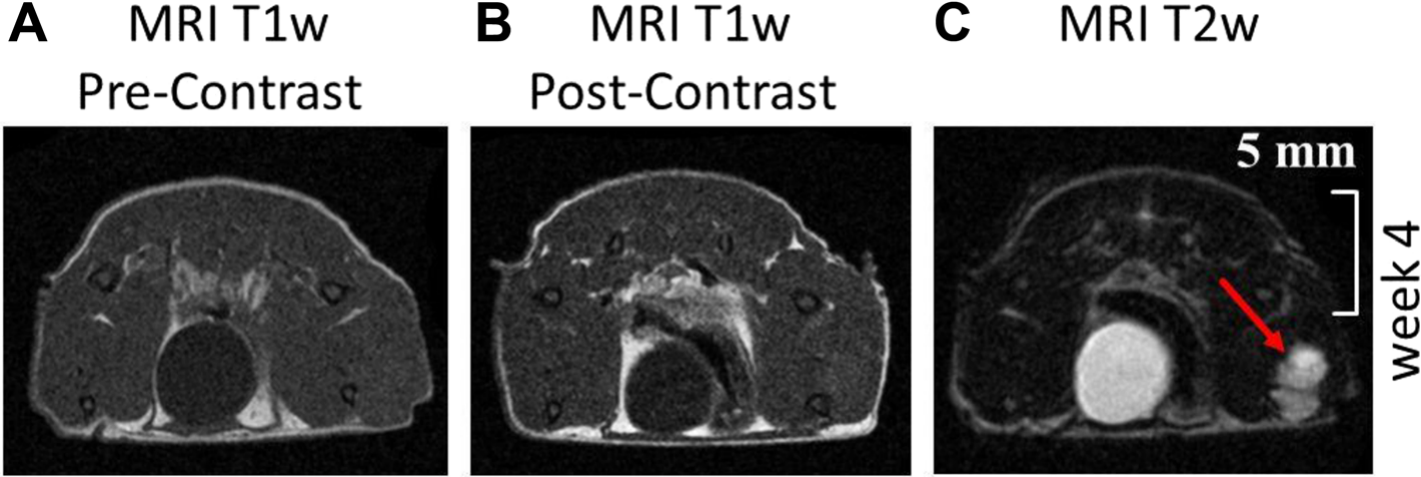
Representative MRI images of T1 weighed (T1w) and T2 weighed (T2w) performed at week 4. **A)** and **B)**: In T1w MRI images pre and post contrast tumor is not visible. **C)**: Using T2w MRI imaging leasion size of 48 μl (here equaling tumor size) can be discriminated (red arrow).

**Figure 3:**
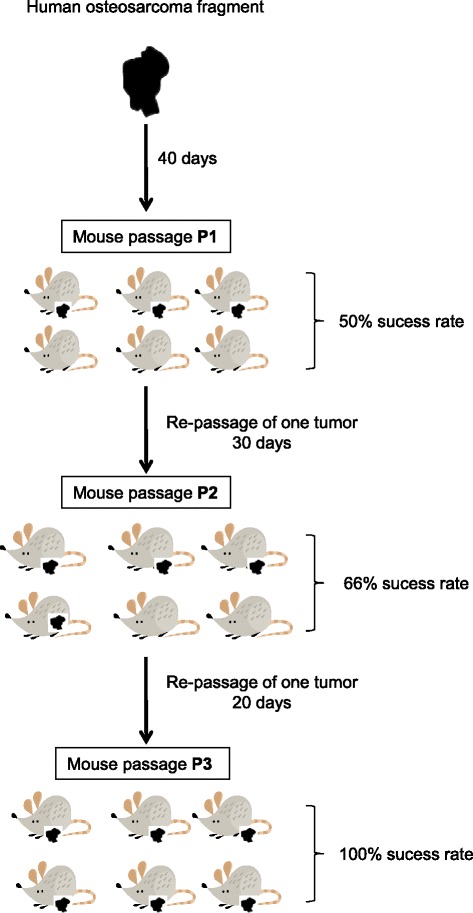
Establishment of patient-derived mouse model. Human OS tissue was transplanted into 6 mice with success rates of 50% in passage 1 (P1), 66% in passage 2 (P2) and 100% in passage 3 (P3). The number of days until tumor onset decreased from 40 (P1) to 30 (P2) to 20 days (P3).

Following biopsy of the relapsed tumor at the right tibia, tumor tissue was transplanted into the right tibia of 6 athymic BALB/c Nu/Nu mice as described in the methods section. After 40 days, 3/6 mice (Passage P1) developed a visible and detectable tumor mass in the MRI which was performed once a week (Figures 1 and 2). The tumors in the 3 mice were removed when the volume reached 1.500 mm^3^. Tumor tissue of one mouse was then re-transplanted immediately into the tibia of 6 further BALB/c Nu/Nu mice (Passage 2). In this passage tumor growth was observed after 30 days in 4 of 6 mice. When the tumor volume reached 1.500 mm^3^, the tumors were removed. One of these tumors was re-transplanted into 6 further mice (Passage 3), which all engrafted after 20 days (Figure 3). At each step, only one tumor was passaged.

The surrounding tissue of all tumors showed local infiltration into the soft tissue as well as the skin. However, until day 60 after tumor implantation into the mice, we could not find metastases anywhere.

### Establishment of a primary OS cell line OS-RH-2011/5

Osteosarcoma tissue from our index patient was directly cultivated (Passage P1*; see methods section). After 26 days, culture was split and 3.75 × 10^6^ cells were injected subcutaneously into the left flank of 6 mice.

**Figure 4:**
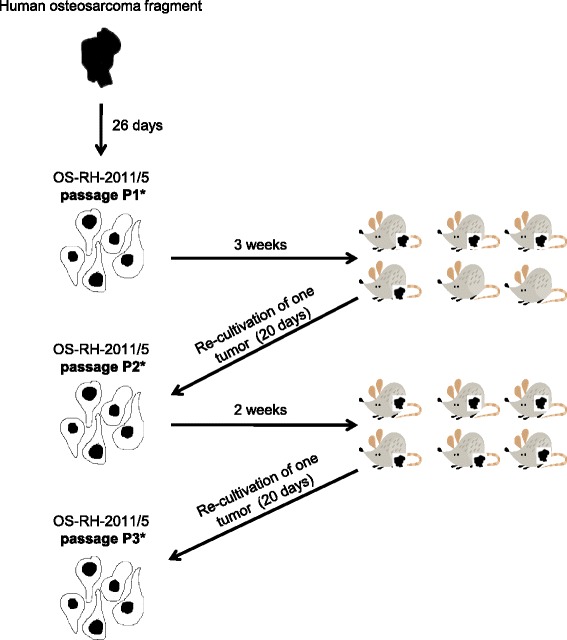
Establishment of the primary tumor cell line OS-RH-2011/5. The original tumor tissue was collected and directly cultivated into medium (P1*). After 26 days, cells were split and 3.75 × 10^6^ cells were injected subcutaneously into the left flank of six athymic BALB/c Nu/Nu mice. After three weeks, four of six mice developed a tumor mass, which was removed when reaching a volume of 1.500 mm^3^. Tumor cells were then passaged in culture (P2*), split after 20 days and again injected into the flank of six mice. After two weeks, all mice developed a tumor mass, which was removed and re-cultured (P3*)

After three weeks, four of six mice developed a tumor mass, which was removed when reaching a volume of 1.500 mm^3^. Tumor cells were then passaged in culture (P2*), split after 20 days and again injected into the flank of six mice. After two weeks, all mice developed a tumor mass, which was removed and re-cultured (P3*). Now, the third culture is used for further experiments e.g. investigation of new treatment strategies (Figure 4). The cells were monitored daily by microscopy. The cell morphology remained stable and very similar to the cells of origin (not shown).

### Genetic and histopathological characterization of the orthotopic patient-derived osteosarcoma xenograft and the primary osteosarcoma cell line

**Figure 5:**
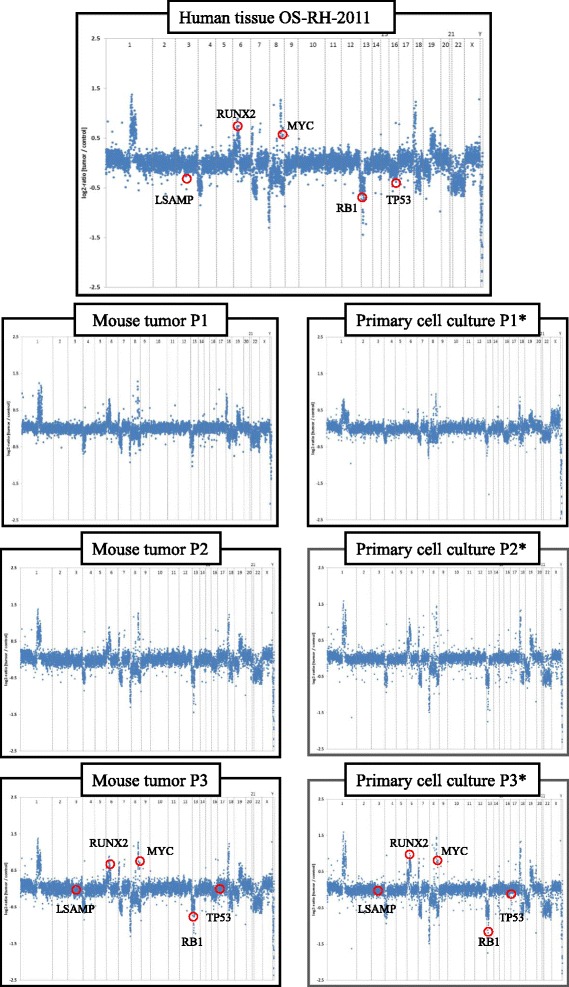
Microarray-based comparative genomic hybridization of the human tumor tissue, the three mice passages and the three cell line passages. Primary orthotopic osteosarcoma xenografts and all cell line passages are genomically stable when compared to the tumor in the patient. The mice tumors and the cell line passages showed similar and for osteosarcoma typical copy number aberrations.

We next analyzed the xenografts and the cell line passages by aCGH to detect genomic copy number variations (CNV) at a high resolution level. As expected for osteosarcoma, this analysis revealed a multitude of CNV. Importantly, all tumors and all cell line passages displayed a very similar genomic pattern, which closely resembled the genomic profile of the primary tumor tissue (Figure 5). Furthermore, we identified identical aberrations which are frequently observed in osteosarcoma (e.g. amplification of Myc and LOH in RB1) [19]. For a detailed overview see Table 2. These findings demonstrate a striking stability of genomic copy number variations in both the orthotopic xenografts as well as in the primary cell line. In addition, we compared the human tissue, the tumor of orthotopic xenografts and subcutaneous tumors. Here, our results showed different genomic patterns of the subcutaneous tumors compared to the human tumor and orthotopic tumors (Table 2). Gains of chromosomes 1, 7, 17 and 19 are not present within the subcutaneous OS model, in contrast an additional loss of regions of chromosome 3 was derived. However, these aberrations are possibly not driver events on OS pathogenesis, since these tumors arise with and without these alterations in different anatomical compartments.

**Table 2:** **Frequent genetic aberrations in osteosarcoma**

**Gene locus (locus name)**	**Human tissue OS-RH-2011**	**Orthotopic mouse tumor P3**	**Subcutaneous mouse tumor P3**	Primary cell cultureP3*
1p22.3 (BCL10)	Gain / amplification	Gain / amplification	No variation	Gain / amplification
3q13.31 (LSAMP)	LOH / Del	LOH / Del	No variation	LOH / Del
3p26.1 (SUMF1)	No variation	No variation	LOH / Del	No variation
6p21.1 (RUNX2)	Gain / amplification	Gain / amplification	No variation	Gain / amplification
7q31.33 (POT1)	Gain / amplification	Gain / amplification	No variation	Gain / amplification
8q24.21 (Myc)	Gain / amplification	Gain / amplification	Gain / amplification	Gain / amplification
13q14 (RB1)	LOH / Del	LOH / Del	LOH / Del	LOH / Del
17p13.1 (TP53)	Gain / amplification	Gain / amplification	No variation	Gain / amplification
17q25.1 (SLC25A19)	Gain / amplification	Gain / amplification	No variation	Gain / amplification
19q13 (GLTSCR1)	Gain / amplification	Gain / amplification	No variation	Gain / amplification

Loss of heterozygocity (LOH) was defined as log2 ratio per clone > − 0.25, Amplification was defined as log2 ratio per clone > 0.25.

**Figure 6:**
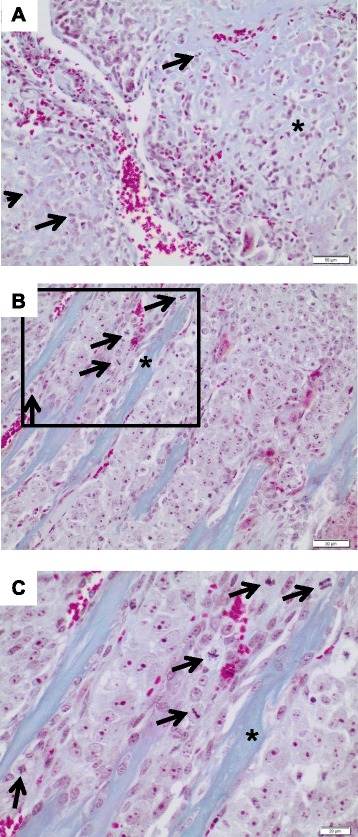
Representative histology of the tumors (Masson trichrom staining). Note the intimate association of neoplastic disorganized, fairly primitive trabeculae (bluish color) with the tumor cells (light red cytoplasm), which either present as lace-like pattern **A)** or as broad sheets **B)**. The arrows indicate mitosis, (*) indicates neoplastic osteoid. **A)**: Relapsed primary osteosarcoma at the time of diagnosis (×20), **B)**: corresponding patient-derived xenograft of mouse passage P3 (×20). As depicted here, the xenograft tumor closely resembles the relapsed primary including the production of neoplastic bone. Note the abundant mitotic tumor cells (arrows). **C)**: Corresponding patient-derived xenograft of mouse passage P3 at higher magnification (framed detail from B; ×40).

Additionally, we investigated the morphology of the primary and our model system. To this end, mouse xenografts were examined by two pathologists. Compared to the clinical biopsies of the primary and relapsed tumors, which were both diagnosed as conventional high grade osteoblastic osteosarcoma, the xenografts displayed similar histological characteristics including mitotic figures and formation of neoplastic extracellular bone matrix into which tumor cells were incorporated (Figure 6).

## Discussion

There is a disappointing lack of progress in the outcome of osteosarcoma patients over the last three decades despite well designed and large international prospective randomized clinical studies [20]. It is particularly notable that osteosarcoma, although occurring in about 5% of children and adolescents with cancer, is now the second most common cause of death in this age group [21]. Therefore, development of new treatment strategies requires systematic preclinical studies that can in turn be translated into the clinic. Existing model systems of osteosarcoma employ subcutaneous injections of immortalized established cell lines into the flank of mice. This model is fast, cheap, reproducible and easy to handle in experimental settings [7,22-28]. However, this model does not sufficiently reflect the human situation.

In 1997, Crnalic et al. reported a novel spontaneous metastasis model of human osteosarcoma developed using orthotopic transplantation of intact tumor cells into tibia of nude mice. Though, they used tumor tissue obtained from the 32nd serial passage of subcutaneously growing human osteosarcoma xenografts but did not perform any further genetic analysis to compare the tumor of origin to the mouse tumor.

In contrast, the important innovation we report here is a technique that enables orthotopic transplantation of human osteosarcoma tissue directly into the tibia of immunodeficient mice resulting in a reproducible and genetically representative patient xenograft model. So far, our model was established successfully using osteosarcoma tissue of three further patients (data not shown). However, the method requires specific technical skill and experience in animal models. But despite the significantly higher technical complexity and higher costs, such patient-derived orthotopic xenografts models can better recapitulate the biology of the human disease and thus facilitating the investigation of novel treatment strategies in a setting that more closely resembles the human primary tumor [29-33]. We are currently employing this novel tool to identify new compounds for the systemic treatment of osteosarcoma and for developing new strategies to achieve local control by heavy ion radiotherapy [34,35].

## Conclusions

In conclusion, we report the first orthotopic osteosarcoma mouse xenograft model, established by transplantation of tumor fragments directly harvested from the patient. This model has been shown to closely reflect the human disease on the level of morphology by MRI scanning and histopathology as well as on the genomic level as revealed by aCGH.
